# A novel deep learning approach to extract Chinese clinical entities for lung cancer screening and staging

**DOI:** 10.1186/s12911-021-01575-x

**Published:** 2021-07-30

**Authors:** Huanyao Zhang, Danqing Hu, Huilong Duan, Shaolei Li, Nan Wu, Xudong Lu

**Affiliations:** 1 College of Biomedical Engineering and Instrument Science, Zhejiang University, Zheda Road, Hangzhou, China; 2 Key Laboratory for Biomedical Engineering, Ministry of Education, Zheda Road, Hangzhou, China; 3 Department of Thoracic Surgery II, Peking University Cancer Hospital & Institute, Beijing, China

**Keywords:** Transformer, BERT, Pre-training, CT reports, Lung cancer screening and staging, Named entity recognition

## Abstract

**Background:**

Computed tomography (CT) reports record a large volume of valuable information about patients’ conditions and the interpretations of radiology images from radiologists, which can be used for clinical decision-making and further academic study. However, the free-text nature of clinical reports is a critical barrier to use this data more effectively. In this study, we investigate a novel deep learning method to extract entities from Chinese CT reports for lung cancer screening and TNM staging.

**Methods:**

The proposed approach presents a new named entity recognition algorithm, namely the BERT-based-BiLSTM-Transformer network (BERT-BTN) with pre-training, to extract clinical entities for lung cancer screening and staging. Specifically, instead of traditional word embedding methods, BERT is applied to learn the deep semantic representations of characters. Following the long short-term memory layer, a Transformer layer is added to capture the global dependencies between characters. Besides, pre-training technique is employed to alleviate the problem of insufficient labeled data.

**Results:**

We verify the effectiveness of the proposed approach on a clinical dataset containing 359 CT reports collected from the Department of Thoracic Surgery II of Peking University Cancer Hospital. The experimental results show that the proposed approach achieves an 85.96% macro-F1 score under exact match scheme, which improves the performance by 1.38%, 1.84%, 3.81%,4.29%,5.12%,5.29% and 8.84% compared to BERT-BTN, BERT-LSTM, BERT-fine-tune, BERT-Transformer, FastText-BTN, FastText-BiLSTM and FastText-Transformer, respectively.

**Conclusions:**

In this study, we developed a novel deep learning method, i.e., BERT-BTN with pre-training, to extract the clinical entities from Chinese CT reports. The experimental results indicate that the proposed approach can efficiently recognize various clinical entities about lung cancer screening and staging, which shows the potential for further clinical decision-making and academic research.

**Supplementary Information:**

The online version contains supplementary material available at 10.1186/s12911-021-01575-x.

## Background

Lung cancer is the most commonly diagnosed cancer and the leading cause of cancer-related deaths, and the situation is particularly urgent in China [1]. Computed tomography (CT), as the primary examination of lung cancer, reports a large volume of valuable information about patients’ conditions and the interpretations from radiologists, which can be used for clinical diagnosis and progression assessment. Besides, the information in clinical narratives was also utilized in many academic studies, e.g., risk evaluation [2, 3], staging [4], decision making [5], and achieved remarkable results. However, the free-text nature of CT reports is a critical barrier to fully use this information [6], and manually extracting structured information from free-text data is time-consuming, error prone, and costly [7].

To extract the information from free-text corpus, Named Entity Recognition (NER) is applied to identify the types and boundaries of interested entities, which has been widely investigated [8]. In earlier studies, rule-based approaches [9, 10] were first proposed to tackle this problem. Although valuable, simplified artificial rules can hardly cover all language phenomena, and intricate rules are difficult to update and maintain and often lead to poor generalization and portability [11]. To alleviate these problems, many researchers turned to machine learning algorithms, e.g., support vector machines (SVM), Conditional Random Fields (CRF), and achieved great power for NER [12–15]. However, the performance of these statistical methods heavily relies on predefined features, which can hardly cover all useful semantic representations for recognition, resulting in poor discriminatory ability of the model [16].

Recently, deep neural network (DNN), especially Recurrent Neural Network (RNN), achieves remarkable performance in Clinical Named Entity Recognition (CNER) tasks. Mostafiz and Ashraf [17] compared the RNN-based NER method with other information extraction tools, e.g., RapTAT [18], MTI [19], in extracting pathological terms from chest X-Ray radiology reports and demonstrated that deep neural network outperformed generic tools by a large margin. Gridach [20] added a CRF layer after the RNN layer to process the CNER task and obtained remarkable results on both JNLPBA and BioCreAtIvE II GM data sets. Zhang et al. [21] used Bi-directional Long Short-Term Memory and Conditional Random Field (BiLSTM-CRF) to automatically identify clinical entities such as diagnosis, symptom, and treatment simultaneously from Chinese Electronic Health Records (EHRs) and achieved better performance than CRF model.

Beside the breakthrough of RNN, recently, self-attention, a special case of attention mechanism, has been widely used to capture richer correlation between words. Unlike RNNs that obtain long dependencies over several time steps [22], which makes it a challenge to learn long-term dependencies when encoding long sequences, self-attention can directly capture long dependencies by calculating the cross interactions between the two tokens in a sentence regardless of their distance [23]. By focusing on some important information, it gives higher weight to important information, while assigning smaller weight to other information received at the same time [16]. Relying entirely on self-attention to draw global dependencies between input and output, Transformer [24] has achieved remarkable performance in a variety of sequence learning tasks [25, 26]. Despite these achievements, it still lacks the components necessary for modeling local structures sequentially and relies heavily on location embeddings that have limited its efficiency [27].

More recently, a novel language representation model, namely Bidirectional Encoder Representations from Transformers (BERT) [28], was proposed by pre-training on large unlabeled corpus using bidirectional transformers. By pre-training Masked Language Model (MLM) and Next Sentence Prediction (NSP) on large plain text corpus, BERT has achieved significant improvement on various Natural Language Processing (NLP) tasks, e.g., NER, Question Answering (QA), Machine Reading Comprehension (MRC), and etc. One of the important applications of BERT is to provide word embedding as features of DNN. As an unsupervised feature learning techniques, word embedding maps the words to vectors of real numbers to capture the semantic and syntactic information between them [29], which has become an indispensable component of DNN for NER tasks. Unlike classical embeddings such as FastText [30] and GloVe that represent the word with polysemy using only one fixed vector, BERT can dynamically adjust the word representation by capturing contextual information and long distant dependencies between words in the sentence [31].

To build a supervised NER model, data annotation is an essential step, but it is expensive and time-consuming [32]. When the labeled data is limited, a lot of linguistic phenomena will not be covered in the training corpus, which may lead to poor generalization of models [33]. Unsupervised pre-training is a popular way to enhance the model performance by learning linguistic phenomena from unlabeled data. In the sense of realizing the minimum of the empirical cost function, unsupervised pre-training can optimally initialize the model’s parameters, thereby somehow making the optimization process more efficient [34].

Although CNER has been extensively studied [17, 20, 21], most of the previous studies did not focus on extracting entities for staging from radiology reports. In this paper, we proposed a novel deep learning approach, namely BERT-based-BiLSTM-Transformer network (BERT-BTN) with pre-training, to extract 14 types of clinical entities from chest CT reports for lung cancer screening and TNM staging. Specifically, BERT was applied as the word embedding layer to learn the word representation. Then, we combined LSTM with Transformer to enjoy the advantages of them while naturally avoid their respective limitations. Specifically, following the traditional LSTM layer, we added a Transformer layer to capture the global dependencies between characters. To alleviate the problem of insufficient labeled data, pre-training technique was employed to initialize the parameters of the proposed model. Experimental results indicate that our method achieves competitive performance for recognizing entities in comparison with benchmark models. To the best of our knowledge, this is the first study to combine those techniques to extract entities from Chinese CT reports for lung cancer screening and TNM staging.

## Methods

### Overview

**Fig. 1:**
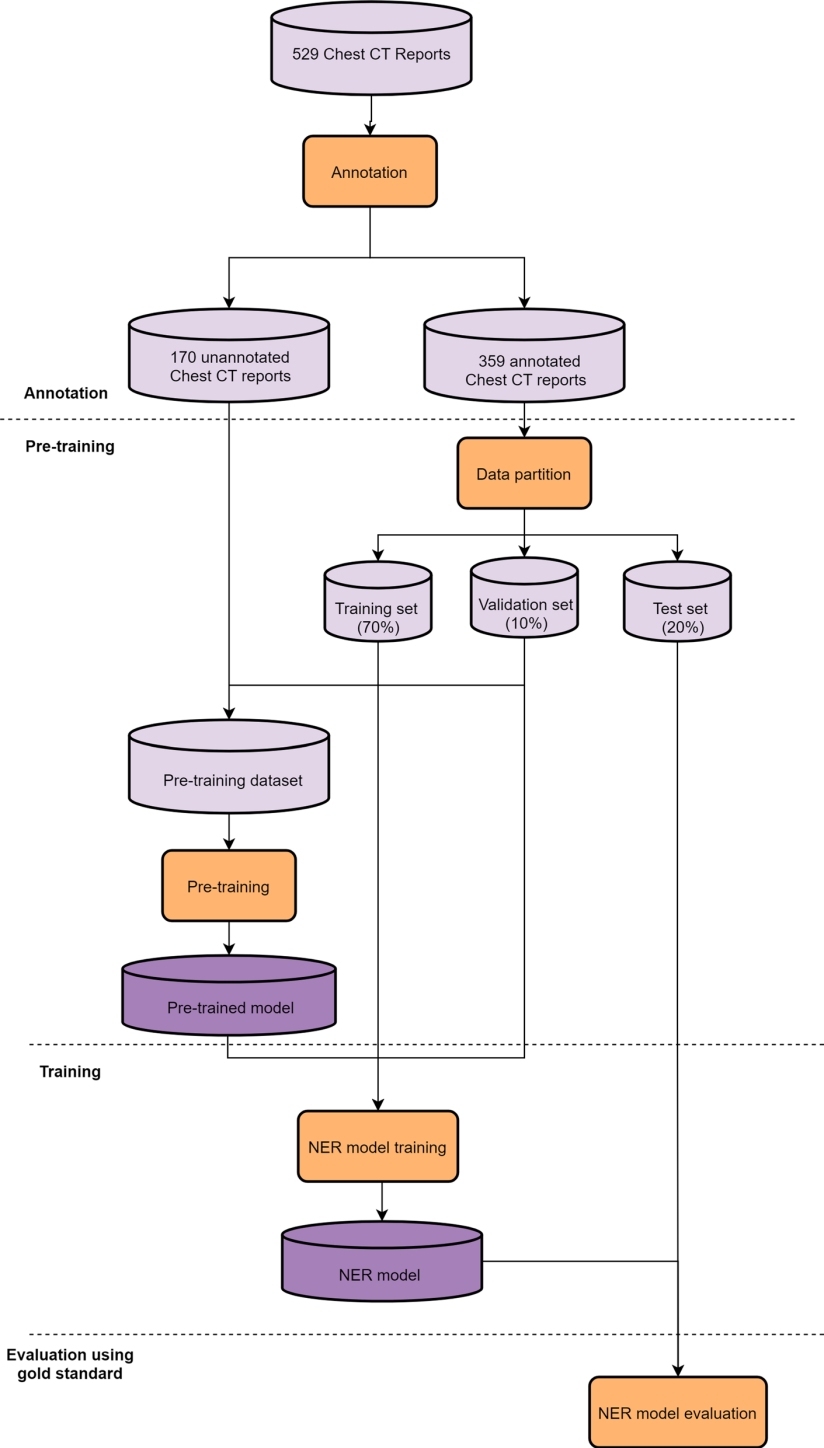
The development pipeline of the proposed method

The development pipeline of the proposed method is shown in Fig. 1. To develop our NER model, we first annotated the pre-defined entities in chest CT reports. And then, the pre-training technique was applied to initialize the parameters of the model. After that, the model was trained, validated, and test on the annotated dataset. The details of the proposed method are elaborated in follows.

### Data and annotation

A total of 529 chest CT reports was collected from the Department of Thoracic Surgery II of Peking University Cancer Hospital. The data contained heterogeneous aspects including patient identification, examination time, findings, conclusion, diagnosis, and etc. In this study, we extracted the information from findings because the information about cancer screening and staging was mainly recorded in findings.

In clinical practice, clinicians usually refer to TNM staging guideline to stage patients. Based on the 8th edition of lung cancer TNM staging guideline [35] and consultations of clinicians at the department, we finally defined a total of 14 types of named entities which covered the screening and staging information in chest CT reports. These entities and corresponding instances are shown in Table 1.

**Table 1:** Entity types for clinical named entity recognition

Entity type	Description	Instance
Vessel	Description of great vessel invasion	病灶包绕右下肺动脉主 (The lesion surrounds the right lower pulmonary trunk)
Vertebral Body	Description of Vertebral Body invasion	颈7椎体压缩变扁(Cervical 7 vertebrae become compressed and flattened)
PAOP^a^	Description of pulmonary atelectasis or obstructive pneumonitis	远端可见片絮影 (Fillets are visible at the far end)
Bronchus	Description of bronchial invasion	凹陷 (indentation)
Pleura	Description of pleural invasion or metastasis	增厚 (thickening)
Shape	Shape of mass	类圆形 (round)
Density	Density of mass	磨玻璃密度 (ground glass density)
Mass	Suspected mass/lump/lesion in lung	结节 (nodule)
Enhancement	Enhancement extent of mass	强化明显 (significant intension)
Size	Size of mass or lymph nodes	25 × 22 cm
Location	Location of mass or lymph nodes	左上肺右基底段 (upper left lung right basal segment)
Lymph	Suspected lymph node metastasis	肿大淋巴结 (swollen lymph nodes)
Negation	Negative words	未见 (no)
Effusion	Condition of pericardial effusion	心包积液 (effusion)

^a^PAOP: Pulmonary Atelectasis/Obstructive Pneumonitis

**Fig. 2:**
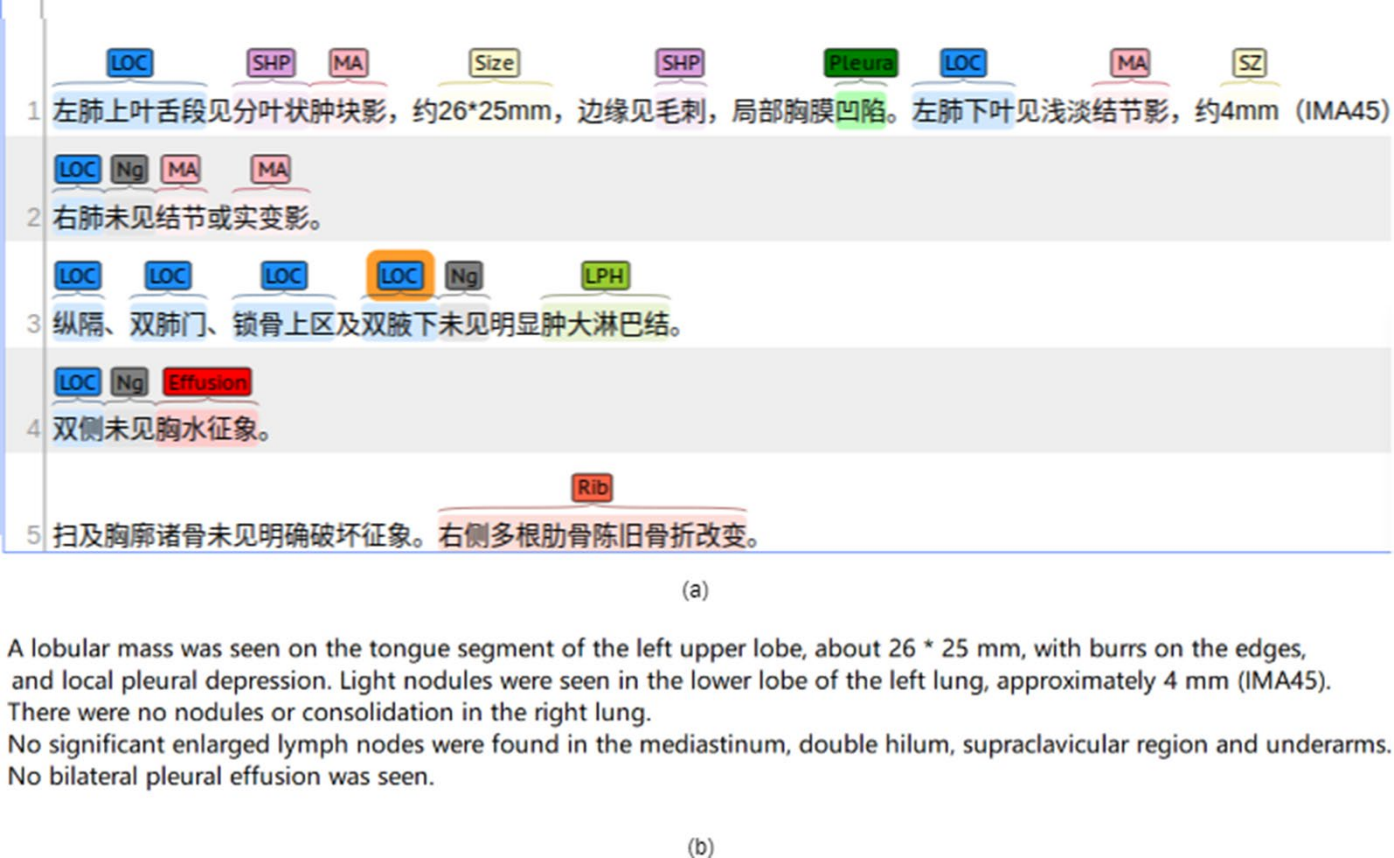
A chest CT report sample annotated with BIO tags **a** Original CT report. LOC: Location; SHP: Shape; MA: Mass; SZ: Size; Ng: Negation; LPH: Lymph. **b** Its’ translation version in English

Based on i2b2 annotation guideline [36] and repeated discussions, we have formulated an annotation guideline, and the annotation guideline is listed in Additional file 1. Two medical informatics engineers were recruited to annotate the chest CT reports manually following the annotation guideline. We used the BIO label scheme, where B, I, and O denote the beginning, inside, and outside characters of an entity, respectively. Figure 2 shows an example of annotated chest CT report. We randomly selected 359 chest CT reports to annotate. The summary statistics of the annotations are shown in Table 2. Then the annotated data was used as the gold standard data to train and evaluate the proposed method. The annotation task was initiated by going through preliminary practice rounds in which annotators were given the same set of 50 CT reports to annotate followed by team meetings where agreement was discussed to clarify ambiguous examples found during preceding practice sessions. Once good understanding of the annotation task was achieved, we selected 100 reports to annotated by both annotators to calculate the inter-rater agreement.

**Table 2:** The statistics of annotated named entities in chest CT reports

Entity type	Total	
	Count	Average length
Vessel	51	10.82
Vertebral Body	28	13.75
PAOP	85	8.77
Bronchus	58	4.66
Pleura	230	4.27
Shape	513	4.37
Density	340	5.00
Mass	874	4.11
Enhancement	185	5.44
Size	774	7.35
Location	1937	8.77
Lymph	588	4.66
Negation	924	4.27
Effusion	412	4.37

### Clinical named entity recognition model

**Fig. 3:**
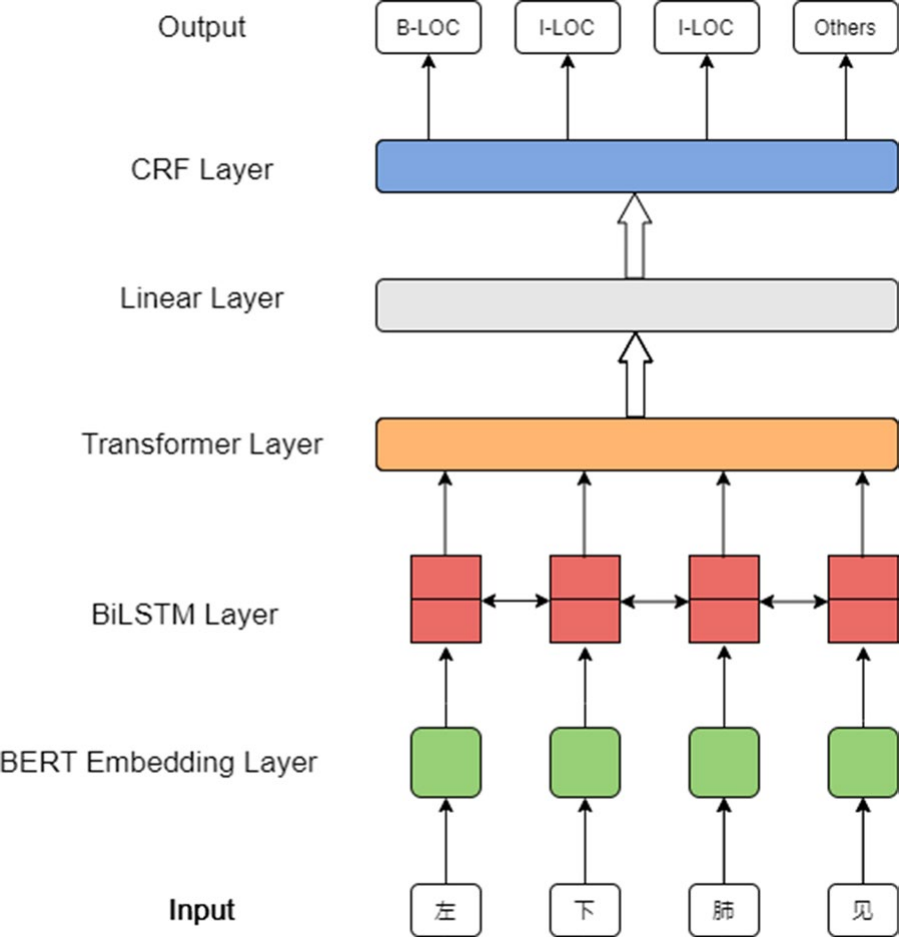
The architecture of the BERT-BTN model

As shown in Fig. 3, given a sentence, we first input the sentence into embedding layer to capture the semantic representation of each character. In this paper, we used the Whole Word Masking version of BERT (BERT-WWM) [37] as the embedding layer, which mitigates the limitations of original BERT by forcing the model to recover the whole word in MLM pre-training task.

Following the word embedding layer, the BiLSTM layer was applied to capture nested structures of the sentence and latent dependency of each character. After that, we used a Transformer layer to draw global dependencies between each character regardless of distance, which can alleviate the burden of the LSTM compressing all relevant information into a single hidden state [38].Then a linear layer was employed to predict possible labels of each character in the sentence. To improve predictive accuracy, we added a CRF layer to learn some constraints from annotated labels to ensure the final predicted labels were valid. Finally, a softmax function was used to output the probabilities of all labels for each character in the sentence.

### Unsupervised pre-training

When the labeled data is limited, pre-training has been proven to effectively improve model performance [39]. In this study, we applied a pre-training method described in the literature [40]. To pre-train the model, we first calculated Term Frequency–Inverse Document Frequency (TF-IDF) vector $TFIDF$ based on all CT reports except those in the test set, the calculation method is shown in Eq. 1.

1 $$\begin{array}{*{20}c} {TFIDF_{w,d} = TF_{w,d} *log\left( {\frac{N}{{DF_{w} }}} \right)\ } \end{array}$$

where ${\text{ d}}$ is a document, w is the word in the document, $TF_{w,d}$ indicates the number of times w occurs in d, N indicates the total number of documents, $DF_{w}$ is the number of documents containing w.

Next, we employed Eq. 2 to normalize the $TFIDF$ as $TFIDF_{normalized}$. Then, we multiplied the $TFIDF_{normalized}$ with its corresponding char embedding $E$ using Eq. 3 to obtain TF-IDF-weighted embedding as the target $y^{*}$ for pre-training. It was shown that these TF-IDF-weighted embeddings were able to capture some of the natural variation between different sentences [40].

2 $$\begin{array}{*{20}c} {TFIDF_{normalized} = \frac{{TFIDF - min\left( {TFIDF} \right)}}{{\max \left( {TFIDF} \right) - min\left( {TFIDF} \right)}}\ } \end{array}$$

3 $$\begin{array}{*{20}c} {y^{*} = TFIDF_{normalized} *E\ } \end{array}$$

To pre-train the model in an unsupervised manner, we used a tanh layer to replace the CRF layer, and the mean-square-error loss to formulate the objective function (Eq. 4).

4 $$\begin{array}{*{20}c} {Loss = \frac{1}{{\text{n}}}\mathop \sum \limits_{i = 1}^{n} \left( {\hat{y}_{i} - y^{*}_{i} } \right)^{2} } \\ \end{array}$$

where ${\text{ n}}$ is the number of words in a sentence, $\hat{y}_{i}$ indicates output of the ith word in the sentence, $y^{*}$ is the corresponding TF-IDF weighted embedding. During pre-training, we only updated parameters of BiLSTM layer, Transformer layer and Linear layer and froze parameters of other layers.

BERT optimizes two training objectives—MLM and NSP. MLM is the task of predicting missing tokens in a sequence from their placeholders. Specifically, it simply masks some percentage of the input tokens at random, and then predicts those masked tokens. In order to train a model that understands sentence relationships, BERT pre-train the NSP task, which takes two sequences ($X_{A}$, ${ }X_{B}$) as input, and predicts whether $X_{B}$ is the direct continuation of $X_{A}$. However, it requires a large collection of unlabeled text to pre-train BERT. Comparing to BERT, our pre-training approach is simpler and doesn’t need so much unlabeled text.

## Experiments and results

To train and evaluate the proposed model, we randomly separated 70% CT reports as the training set, 10% as the validation set, and 20% as the test set. To determine the optimal hyper-parameters, a grid search was applied to the training set. Our hyper-parameter spaces are Learning_Rate $\in${1e^−4^,5e^−4^,1e^−3^,5e^−3^}, Dropout $\in${0,0.1,0.2,0.3,0.4,0.5}, Batch_Size $\in${8,16}, LSTM_Laye $\in${1,2}, LSTM_Hidden_Size $\in${64,128}, Transformer_Layer $\in${1,2,3,5}, Transformer_Head $\in${1,2,3,4,6,8,12}. The hyper-parameters used in this paper are listed in Table 3. The standard back-propagation was used to update all parameters and Adam algorithm [41] was employed to optimize the objective function. To avoid overfitting problem, an early stopping strategy [42] was employed on the validation set.

**Table 3:** The main hyper-parameters for the proposed model

Parameter	Setting
LSTM_Hidden_Size	128
LSTM_Layer	1
Transformer_Layer	1
Transformer_Head	1
Dropout	0.13
Batch_size	8
Learning_Rate	1e−4

Two evaluation scoring schemes were used, i.e., exact match and inexact match, where exact match scheme only counts perfect matches when compared to the gold standard; the inexact match means entity is correctly predicted if it overlaps with the corresponding entity in the gold standard. We selected precision, recall, and F1 score as evaluation metrics to measure the performance of our model.

To investigate the effectiveness of the proposed approach, extensive experiments were carried out over the collected data including (1) replacing BERT embedding with FastText embedding, (2) removing transformer layer from the proposed model, (3) removing BiLSTM layer from the proposed model, (4) canceling pre-training step, (5) directly fine-tuning with BERT. We ran our experiments five times and averaged the 5 results as the final result to reduce the possible bias from dataset partitioning.

Based on the annotated 100 reports by the two annotators, the inter-annotation agreement using kappa statistics [43] is 0.937, which indicates the annotation is reliable. Table 4 shows the overall performance of the proposed and benchmark models. As shown in Table 4, the BERT-BTN with pre-training achieves the best performance with 85.96% macro-F1 score and 90.67% micro-F1 score under the exact match scheme and 94.56% macro-F1 score and 96.78% micro-F1 score under the inexact match scheme in comparison with the benchmark models.

**Table 4:** The f1 scores of the proposed and benchmark models

Model	Inexact-match		Exact-match	
	Macro	Micro	Macro	Micro
FastText-Transformer	89.29 ± 2.64	95.25 ± 0.46	77.12 ± 4.14	86.85 ± 1.18
FastText-BiLSTM	90.46 ± 1.31	95.72 ± 0.70	80.67 ± 0.87	88.08 ± 1.41
FastText-BTN	90.47 ± 1.82	95.22 ± 0.52	80.84 ± 3.16	87.76 ± 1.30
BERT-Transformer	90.94 ± 0.69	95.80 ± 0.31	81.67 ± 6.14	87.35 ± 1.23
BERT-BiLSTM	93.05 ± 0.89	97.27 ± 0.16	84.12 ± 1.59	90.13 ± 0.92
BERT- BTN	94.40 ± 0.91	**97.28 ± 0.60**	84.58 ± 2.72	**90.78 ± 1.04**
BERT-fine-tune	92.43 ± 0.61	96.22 ± 0.93	82.15 ± 3.41	88.33 ± 3.00
BERT-BTN (with pre-training)	**94.56 ± 0.80**	96.78 ± 0.73	**85.96 ± 0.46**	90.67 ± 0.51

Bold value indicates the values is best score in the current evaluation index

To prove the effectiveness of BERT embedding, we selected the FastText embedding, a classical embedding that represents the word using only one fixed vector, as the baseline. By analyzing the performances of these two word embedding methods, we can notice that models using BERT embedding outperform models using FastText embedding with an improvement of 4.55% macro-F1 score under exact match scheme and 3.93% macro-F1 score under inexact match scheme at most. The performance improvements indicate BERT is more powerful in contextual information encoding by taking both left and right contexts of target words into account.

BERT-BTN provides 0.46% overall performance improvement under exact match scheme and 1.35% under inexact match scheme compared with BERT-BiLSTM, indicating the long-term dependencies learnt by Transformer are useful for NER. When comparing BERT-Transformer with BERT-BTN, the macro-F1 score drops by 2.91% under exact match scheme and 3.46% under inexact match scheme, indicating the position information encoded by BiLSTM has a significant influence on Transformer’s performance. The reason for performance reduction may be that Transformer only relies on self-attention to draw global dependencies of input and treats every position identically, which may neglect some fixed patterns in the sentences since some information is described by several clauses in a fixed order.

**Fig. 4:**
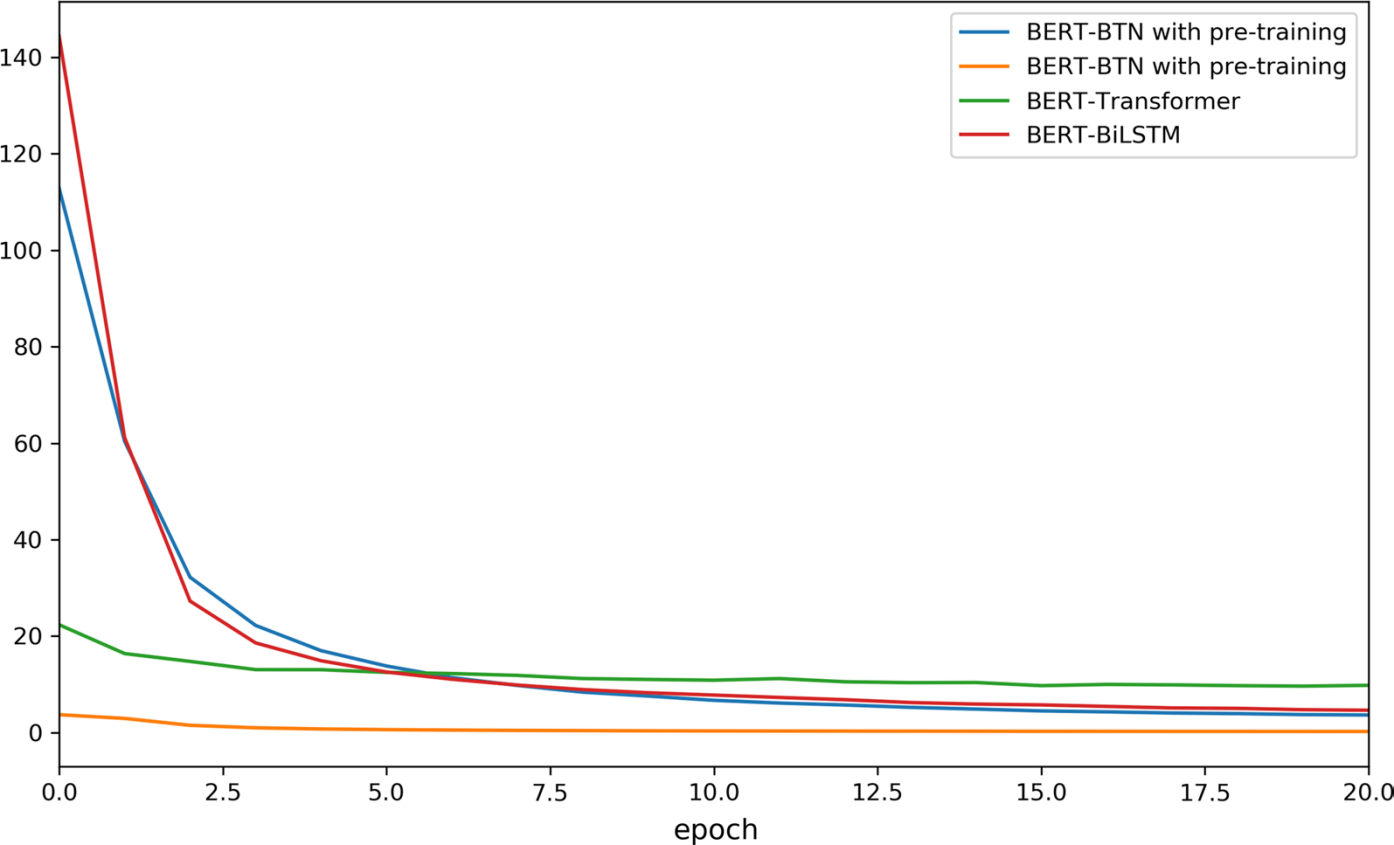
The training loss of models using BERT embedding

Also, we directly fine-tuned BERT and the result shows the simple fine-tuned BERT cannot achieve competitive performances under both exact and inexact match scheme in comparison with the other BERT-based models, indicating that it remains a challenge to achieve good results by fine-tuning BERT directly on some domain-specific tasks. Moreover, when applying the pre-training technique, both prediction accuracy and the speed of convergence gain considerable improvements in comparison with BERT-BTN. As depicted in Fig. 4, using TF-IDF–weighted character embeddings to pre-train the model can almost optimally initialize the model’s parameters so as to accelerate convergence.

Table 5 shows the macro-F1 score of each type of entities under exact match scheme. As shown in Table 5, all models achieved competitive performances and over 90% macro-F1 scores for recognizing Size type of entities, Effusion type of entities, Lymph type of entities, Negation type of entities, and Size type of entities. For complex entities with various expression types, i.e., PAOP type of entities, Vessel type of entities, and Pleura type of entities, the performances were significantly different between different models. Specifically, BERT provided the most improvement because it can dynamically adjust embeddings according to the current context to capture more meaningful semantic information. For instance, we notice that some abnormal tokens such as “ 增厚(thickening)”, “凹陷 (indentation)”, “截断 (truncated)” are labeled as Pleura type of entities or Bronchus type of entities based on different contexts, BERT can provide different embeddings for the same token depending on its context so that the BERT-based models can achieve better results. Besides, the self-attention mechanism can bring some benefits to recognize complex entities due to the ability to capture the global long dependencies and maximize the useful context-related information in the resource. Moreover, for the two longest entity types, i.e., Vertebral Body type of entities and Vessel type of entities, pre-training leads to significant improvements on the macro-F1 score from 70.67% to 82.41%, 59.05% to 65.95%, respectively. Since pre-training can obtain more general linguistic phenomena from unlabeled text, which can provide some benefit for the model to identify long entities.

**Table 5:** The exact match macro-f1 scores of the proposed and benchmark models about 14 types of entities

Entity type	FastText-Transformer	FastText-BiLSTM	FastText-BTN	BERT-Transformer	BERT-BiLSTM	BERT- BTN	BERT-fine-tune	BERT-BTN (pre-training)
Vessel	58.01 ± 11.71	54.54 ± 3.20	56.63 ± 13.56	47.42 ± 14.93	58.48 ± 5.28	59.05 ± 0.51	57.31 ± 18.21	**65.95 ± 7.63**
Vertebral Body	62.67 ± 24.11	59.01 ± 22.65	65.02 ± 12.48	74.41 ± 21.69	63.70 ± 24.61	70.67 ± 16.36	55.71 ± 35.11	**82.41 ± 10.86**
PAOP	60.54 ± 11.24	62.43 ± 8.84	66.94 ± 10.75	55.27 ± 18.44	75.49 ± 9.25	74.50 ± 12.46	72.32 ± 11.29	**78.97 ± 11.53**
Bronchus	60.55 ± 7.26	72.01 ± 3.48	70.79 ± 6.93	76.30 ± 7.61	79.81 ± 9.71	80.15 ± 5.64	**82.13 ± 5.24**	79.37 ± 4.06
Pleura	66.71 ± 10.56	84.22 ± 7.32	82.46 ± 7.53	79.64 ± 7.51	**85.61 ± 5.83**	84.30 ± 2.60	85.10 ± 6.41	85.57 ± 3.87
Shape	69.90 ± 7.34	77.52 ± 2.95	73.56 ± 2.64	79.25 ± 9.93	**82.00 ± 3.40**	80.69 ± 2.77	81.65 ± 1.79	**82.00 ± 2.99**
Density	84.33 ± 1.19	81.37 ± 3.23	83.85 ± 1.65	85.49 ± 8.00	87.46 ± 2.51	**88.75 ± 2.55**	87.21 ± 4.56	86.08 ± 1.88
Mass	80.16 ± 2.28	82.35 ± 3.16	83.04 ± 2.11	84.99 ± 7.43	84.76 ± 2.72	85.13 ± 2.4**7**	77.44 ± 6.07	**85.41 ± 3.78**
Enhancement	74.51 ± 5.76	80.66 ± 2.81	76.54 ± 10.95	**87.70 ± 7.77**	85.29 ± 5.76	84.33 ± 7.36	80.24 ± 14.90	84.27 ± 6.36
Size	93.30 ± 1.65	95.58 ± 1.09	95.58 ± 1.08	95.70 ± 4.59	95.63 ± 1.78	**96.05 ± 1.39**	96.03 ± 0.87	95.70 ± 1.32
Location	83.87 ± 6.41	86.84 ± 2.46	86.87 ± 1.65	89.00 ± 3.58	91.36 ± 0.66	**91.59 ± 0.9**7	88.55 ± 4.00	90.60 ± 2.54
Lymph	90.51 ± 4.00	**94.30 ± 2.34**	94.16 ± 3.24	93.13 ± 1.17	93.65 ± 7.06	93.60 ± 3.66	94.09 ± 2.26	91.98 ± 3.46
Negation	98.56 ± 0.41	**98.97 ± 0.40**	98.58 ± 0.58	98.45 ± 2.97	98.84 ± 0.39	98.30 ± 0.22	94.59 ± 8.53	98.79 ± 0.38
Effusion	96.12 ± 1.72	97.84 ± 0.18	97.82 ± 1.07	96.62 ± 4.10	95.61 ± 3.47	**98.01 ± 1.80**	97.78 ± 0.92	96.52 ± 0.48

Bold value indicates the values is best score in the current evaluation index

## Discussion

In this study, we proposed a novel deep learning method, namely BERT-BTN with pre-training, to recognize 14 types of clinical entities from chest CT reports for lung cancer screening and TNM staging. The results illustrated in Tables 4 and 5 indicate that models with BERT embedding obtains a significant improvement compared with models with FastText embedding. Besides, Transformer provides overall performance improvement and positional information has an important impact for Transformer-based models to recognize entities. Pre-training gain significant improvements in both recognition accuracy and the speed of converge. Also, fine-tuning BERT directly on some domain-specific tasks may not achieve so satisfactory results. The experimental results indicate that the proposed method can efficiently recognize various clinical entities about lung cancer screening and staging, which shows the potential for further clinical decision-making and academic research.

Although the proposed method achieves competitive overall performance for the NER task, it should be mentioned that there are some limitations in our work.

First, we should notice that some types of entities are still not accurately recognized. As shown in Tables 5 and 6, the Vessel type of entities is not recognized satisfactorily like the other types of entities. The first reason may be the number of Vessel type of entities is small, so that an inaccurate recognition can significantly reduce its accuracy. Secondly, the average length of Vessel type of entities is much longer and its pattern is more complex than the other entities, which make it difficult to identify the entity boundaries. When the Vessel type of entities contain some other types of entities that appear frequently like Mass type of entities and Location type of entities, it is a challenge for the model to exactly recognize the whole Vessel entity. For instance, the phrase “右肺动脉分支局限性管腔变窄 (The lumen of the right pulmonary artery branch narrowed)” was annotated as the Vessel type of entities, while our model identified the token “狭窄(narrowed)” in this phase as Bronchus type of entities. One straightforward approach is to get more labeled data containing entities mentioned above to train our model. Zhao et al. [44] showed that training on a specific domain dataset provided better performance than training on a large, general domain dataset. Moreover, using more Chinese clinical corpus to train the Bert-based embedding may be another way to improve the recognition performances of long and complex entities.

**Table 6:** The inexact match macro-f1 scores of the proposed and benchmark models about 14 types of entities

Entity type	FastText-Transformer	FastText-BiLSTM	FastText-BTN	BERT-Transformer	BERT-BiLSTM	BERT- BTN	BERT-fine-tune	BERT-BTN (pre-training)
Vessel	68.74 ± 5.78	59.25 ± 8.77	62.63 ± 9.30	51.06 ± 13.44	67.00 ± 9.22	73.48 ± 12.46	70.74 ± 10.08	**77.25 ± 6.73**
Vertebral Body	91.81 ± 7.22	92.75 ± 6.39	**93.81 ± 8.52**	85.95 ± 6.79	80.00 ± 27.39	85.79 ± 11.08	85.24 ± 10.16	91.69 ± 13.64
PAOP	76.25 ± 7.62	73.30 ± 14.43	77.31 ± 14.70	83.17 ± 4.08	91.16 ± 1.23	93.80 ± 3.13	85.44 ± 10.16	**93.93 ± 2.26**
Bronchus	73.86 ± 9.92	85.79 ± 6.32	83.21 ± 5.63	83.74 ± 3.51	**93.27 ± 3.88**	90.54 ± 3.49	89.67 ± 4.51	91.83 ± 3.00
Pleura	85.45 ± 7.95	93.70 ± 3.08	91.71 ± 4.50	84.78 ± 3.55	96.17 ± 1.77	95.25 ± 2.62	94.36 ± 4.48	**96.20 ± 2.24**
Shape	87.30 ± 6.44	89.73 ± 1.43	88.04 ± 2.74	89.01 ± 1.09	**94.51 ± 1.71**	93.96 ± 2.10	93.28 ± 2.13	92.41 ± 1.74
Density	91.68 ± 1.34	91.06 ± 1.77	92.72 ± 1.01	92.45 ± 1.83	93.34 ± 1.16	**96.17 ± 2.36**	94.86 ± 2.81	95.49 ± 0.78
Mass	95.32 ± 2.39	96.20 ± 1.11	96.83 ± 1.11	94.34 ± 1.06	97.01 ± 0.61	**97.93 ± 0.84**	95.38 ± 3.96	96.86 ± 0.75
Enhancement	89.62 ± 5.16	92.68 ± 3.00	89.04 ± 7.50	92.74 ± 1.89	95.48 ± 3.98	95.28 ± 3.2	94.51 ± 4.86	**95.58 ± 2.98**
Size	98.61 ± 0.46	98.44 ± 0.83	98.34 ± 0.46	97.74 ± 0.74	**99.04 ± 0.62**	99.03 ± 0.59	98.45 ± 0.55	98.62 ± 0.77
Location	93.52 ± 2.18	95.33 ± 1.05	95.48 ± 0.48	93.77 ± 0.92	**97.46 ± 0.53**	97.41 ± 0.61	94.51 ± 3.35	97.24 ± 2.08
Lymph	99.58 ± 0.37	**99.78 ± 0.18**	99.71 ± 0.30	95.85 ± 2.54	99.13 ± 0.28	98.25 ± 1.76	99.41 ± 0.57	98.60 ± 2.28
Negation	98.88 ± 0.36	99.06 ± 0.28	98.66 ± 0.52	99.05 ± 0.14	**99.08 ± 0.30**	98.97 ± 0.25	99.00 ± 0.25	98.88 ± 0.11
Effusion	**99.51 ± 0.28**	99.31 ± 0.59	99.09 ± 0.64	97.65 ± 1.01	99.05 ± 1.31	99.08 ± 0.79	99.18 ± 0.61	99.30 ± 1.00

Bold value indicates the values is best score in the current evaluation index

**Fig. 5:**
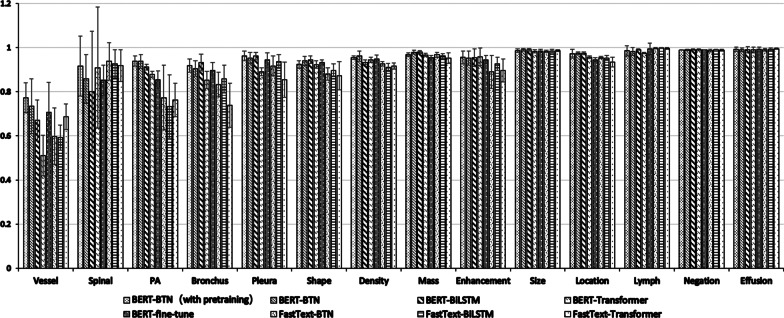
Comparison of the proposed and benchmark models about 14 types of named entities under exact match scheme

**Fig. 6:**
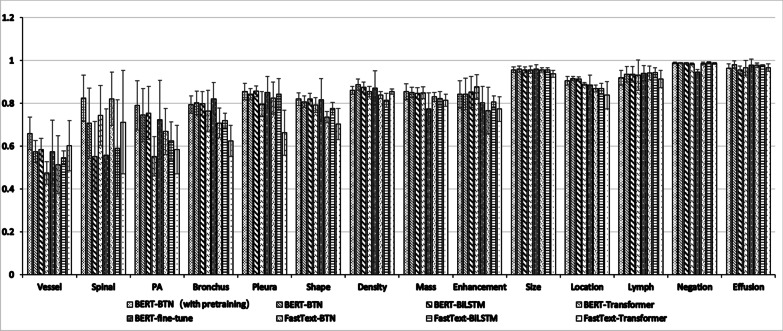
Comparison of the proposed and benchmark models about 14 types of named entities under inexact match scheme

Second, as shown in Tables 5 and 6 and Figs. 5 and 6, the different performances under inexact match scheme and exact match scheme indicate some entities, e.g., Vessel type of entities, Vertebral Body type of entities, were only recognized partially. Yu et al. [45] presented a model which labeled the start and end positions separately in a cascade structure and decoded them together by a multi-span decoding algorithm. They found that predicting end positions might benefit from the prediction results of start positions, which may help to narrow the gap between exact match and inexact match. In the future, we can also try this strategy to explore whether it can further improve the performance.

## Conclusion

In this paper, we proposed a novel deep learning method, namely the BERT-BTN with pre-training, to extract 14 types of clinical entities from Chinese chest CT reports for lung cancer screening and TNM staging. The experimental results show that our model outperforms the benchmark BERT-BTN, BERT-LSTM, BERT-fine-tune, BERT-Transformer, FastText-BTN, FastText-BiLSTM and FastText-Transformer models and achieves the best macro-F1 score of 85.96%, which shows great potential for further utilization in clinical decision support and academic research.

## Supplementary Information

**Additional file 1**: A guideline for annotating 14 types of clinical entities from chest CT reports for lung cancer screening and TNM staging.

## Data Availability

The datasets generated and/or analyzed during the current study are not publicly available due to the hospital’s regulations, but are available from the corresponding author on reasonable request.

## Abbreviations

BiLSTM: Bi-directional long short-term memory
BERT: Bidirectional encoder representations from transformers
BTN: BiLSTM-transformer network
CNER: Clinical named entity recognition
CRF: Conditional random fields
CT: Computed tomography
DNN: Deep neural network
EHRs: Electronic health records
MLM: Masked language model
NER: Named entity recognition
NLP: Natural language processing
NSP: Next sentence prediction
MRC: Machine reading comprehension
QA: Question answering
RNN: Recurrent neural network
SVM: Support vector machines
TF-IDF: Term frequency-inverse document frequency

## References

[1] Lu S, Yu Y, Yang Y (2019). Retrospect and prospect for lung cancer in China: clinical advances of immune checkpoint inhibitors. Oncologist.

[2] Hu D, Huang Z, Chan T, Dong W, Lu X, Duan H (2016). Utilizing Chinese admission records for MACE prediction of acute coronary syndrome. Int J Environ Res Public Health.

[3] Hu D, Li S, Huang Z, Wu N, Lu X (2020). Predicting postoperative non-small cell lung cancer prognosis via long short-term relational regularization. Artif Intell Med.

[4] Risko R, Merdan S, Womble PR, Barnett C, Ye Z, Linsell SM, Montie JE, Miller DC, Denton BT (2014). Clinical predictors and recommendations for staging computed tomography scan among men with prostate cancer. Urology.

[5] Dohan D, Garrett SB, Rendle KA, Halley M, Abramson C (2016). The importance of integrating narrative into health care decision making. Health Affair.

[6] Thomas AA, Zheng C, Jung H, Chang A, Kim B, Gelfond J, Slezak J, Porter K, Jacobsen SJ, Chien GW (2014). Extracting data from electronic medical records: validation of a natural language processing program to assess prostate biopsy results. World J Urol.

[7] Meystre SM, Savova GK, Kipperschuler KC, Hurdle JF (2008). Extracting information from textual documents in the electronic health record: a review of recent research. Yearb Med Inform.

[8] Magge A, Scotch M, Gonzalez-Hernandez G. Clinical NER and relation extraction using bi-char-LSTMs and random forest classifiers. In: International workshop on medication and adverse drug event detection. 2018: p. 25–30.

[9] Nguyen AN, Lawley MJ, Hansen DP, Bowman RV, Clarke BE, Duhig EE, Colquist S (2010). Symbolic rule-based classification of lung cancer stages from free-text pathology reports. J Am Med Inform Assoc.

[10] Chen L, Song L, Shao Y, Li D, Ding K (2019). Using natural language processing to extract clinically useful information from Chinese electronic medical records. Int J Med Inform.

[11] Nasar Z, Jaffry SW, Malik MK (2018). Information extraction from scientific articles: a survey. Scientometrics.

[12] Nandhakumar N, Sherkat E, Milios EE, Gu H, Butler M. Clinically significant information extraction from radiology reports. In: Proceedings of the 2017 ACM symposium on document engineering. 2017: p. 153–162.

[13] Soysal E, Warner JL, Denny JC, Xu H (2017). Identifying Metastases-related Information from pathology reports of lung cancer patients. Amia Jt Summits Transl Sci Proc.

[14] Hassanpour S, Langlotz CP (2016). Information extraction from multi-institutional radiology reports. Artif Intell Med.

[15] Warner JL, Levy MA, Neuss MN, Warner JL, Levy MA, Neuss MN (2016). ReCAP: feasibility and accuracy of extracting cancer stage information from narrative electronic health record data. J Oncol Pract.

[16] Liao F, Ma L, Pei J, Tan L (2019). Combined self-attention mechanism for Chinese named entity recognition in military. Future Internet.

[17] Mostafiz T, Ashraf K. Pathology extraction from chest X-ray radiology reports: a performance study. arXiv:1812.02305 2018.

[18] Gobbel GT, Garvin J, Reeves R, Cronin RM, Heavirland J, Williams J, Weaver A, Jayaramaraja S, Giuse D, Speroff T (2014). Assisted annotation of medical free text using RapTAT. J Am Med Inform Assoc.

[19] Aronson AR, Mork J, Lang F, Rogers W, Jimeno-Yepes A, Sticco JC (2012). The NLM indexing initiative: current status and role in improving access to biomedical information.

[20] Gridach M (2017). Character-level neural network for biomedical named entity recognition. J Biomed Inform.

[21] Zhang Y, Wang X, Hou Z, Li J (2018). Clinical Named Entity Recognition From Chinese Electronic Health Records via Machine Learning Methods. JMIR Med Inform.

[22] Zhou P, Shi W, Tian J, Qi Z, Li B, Hao H, Xu B. Attention-based bidirectional long short-term memory networks for relation classification. In: Proceedings of the 54th annual meeting of the association for computational linguistics (Volume 2: Short Papers). 2016, p. 207–212.

[23] Tang G, Müller M, Rios A, Sennrich R. Why self-attention? a targeted evaluation of neural machine translation architectures. arXiv:1808.08946 2018.

[24] Vaswani A, Shazeer N, Parmar N, Uszkoreit J, Jones L, Gomez AN, Kaiser A, Polosukhin I. Attention is all you need. In: Advances in neural information processing systems. 2017, p. 5998–6008.

[25] Bai S, Kolter JZ, Koltun V. An empirical evaluation of generic convolutional and recurrent networks for sequence modeling. arXiv:1803.01271 2018.

[26] Gehring J, Auli M, Grangier D, Yarats D, Dauphin YN. Convolutional sequence to sequence learning. arXiv:1705.03122 2017.

[27] Wang Z, Ma Y, Liu Z, Tang J. R-transformer: Recurrent neural network enhanced transformer. arXiv:1907.05572 2019.

[28] Devlin J, Chang MW, Lee K, Toutanova K: BERT: Pre-training of deep bidirectional transformers for language understanding. arXiv:1810.04805 2018.

[29] Mikolov T, Chen K, Corrado G, Dean J. Efficient estimation of word representations in vector space. arXiv:1301.3781 2013.

[30] Bojanowski P, Grave E, Joulin A, Mikolov T (2017). Enriching Word Vectors with Subword Information. Trans Assoc Comput Linguist.

[31] Si Y, Wang J, Xu H, Roberts K. Enhancing clinical concept extraction with contextual embedding. arXiv:1902.08691 2019. 10.1093/jamia/ocz096 PMC6798561 31265066

[32] Erhan D, Bengio Y, Courville A, Manzagol PA, Vincent P, Bengio S. Why does unsupervised pre-training help deep learning? In: Proceedings of the thirteenth international conference on artificial intelligence and statistics. 2010, p. 201–208.

[33] Johnson R, Zhang T. Supervised and semi-supervised text categorization using LSTM for region embeddings. arXiv:1602.02373 2016.

[34] Bengio Y, LeCun Y (2007). Scaling learning algorithms towards AI. Large-Scale Kernel Mach.

[35] Rami-Porta R, Goldstraw P, Pass HI. The eighth edition of the tumor, node, and metastasis classification of lung cancer. IASLC Thoracic Oncology. Content Repository Only!, 2018, p. 253–264.

[36] i2b2/VA Challenge. Concept Annotation Guidelines. 2010. https://www.i2b2.org/NLP/Relations/assets/ConceptAnnotation Guideline.pdf. Accessed 11 Nov 2016.

[37] Cui Y, Che W, Liu T, Qin B, Yang Z, Wang S, Hu G. Pre-training with whole word masking for Chinese BERT. arXiv:1906.08101 2019.

[38] Li P, Ma W. Understanding and improving sequence-labeling NER with self-attentive LSTMs. 2018.

[39] Kale M, Siddhant A, Nag S, Parik R, Grabmair M, Tomasic A. Supervised contextual embeddings for transfer learning in natural language processing tasks. arXiv:1906.12039 2019.

[40] Gao S, Young MT, Qiu JX, Yoon H, Christian JB, Fearn PA, Tourassi GD, Ramanthan A (2017). Hierarchical attention networks for information extraction from cancer pathology reports. J Am Med Inform Assoc.

[41] Kingma DP, Ba J. Adam: A method for stochastic optimization. arXiv:1412.6980 2014.

[42] Prechelt L (1998). Automatic early stopping using cross validation: quantifying the criteria. Neural Netw.

[43] Hripcsak G, Rothschild AS (2005). Agreement, the f-measure, and reliability in information retrieval. J Am Med Inform Assoc.

[44] Zhao M, Masino AJ, Yang CC. A framework for developing and evaluating word embeddings of drug-named entity. In: Proceedings of the BioNLP 2018 workshop. 2018, p. 156–160.

[45] Yu B, Zhang Z, Su J. Joint extraction of entities and relations based on a novel decomposition strategy. arXiv:1909.04273 2019.

