# Supplementary material for: A novel deep learning approach to extract Chinese clinical entities for lung cancer screening and staging
Source: BMC Med Inform Decis Mak. 2021 Jul 30;21(Suppl 2):214. doi: 10.1186/s12911-021-01575-x (PMC8323233; doi:10.1186/s12911-021-01575-x)
Supplement: Supplementary file 1 — Additional file 1: A guideline for annotating 14 types of clinical entities from chest CT reports for lung cancer screening and TNM staging. [file 12911_2021_1575_MOESM1_ESM.pdf]

Annotation guideline

General Guidelines for Annotation

- Punctuation should not be annotated separately, and is not included in the annotation as far as possible. This is to minimize the interference of symbols on the meaning of annotated entities.

- Entity annotation is not overlapped or nested. The same object should not be annotated as different entities.

- For some complex or ambiguous situations, we may make some special appointments based on the annotation types and doctors' suggestions, similar objects will be annotated according to these appointments.

- The benign words won't be annotated, such as "边界清楚(clear edges)", "右侧胸膜光滑(smooth right pleura)".

- If there is a conjunction in the entity, e.g., "边缘可见分叶并多发毛刺 (lobulated and multiple burred margins)", "分叶 (lobulated)" and "多发毛刺 (multiple burred)" are supposed to be annotated as one entity (one Shape entity).

Mass

This type of entity refers to descriptions of suspicious masses

Several typical examples are described in the following:

- 左肺下叶基底段见不规则软组织团块[there were irregular soft tissue masses in the basal segment of the left lower lobe] = 软组织团块("soft tissue masses").

- 右肺上叶前段可见一分叶状软组织密度结节[there were lobular soft tissue density nodules in the anterior segment of the right upper lobe] = 软组织密度结节("soft tissue density nodules").

Lymph

This type of entity refers to descriptions of suspicious lymph nodes metastasis

Several typical examples are described in the following:

- 纵隔 2R、4、7 组多发肿大淋巴结[there were multiple enlarged lymph nodes in mediastinal 2R, 4, and 7 groups] = 肿大淋巴结(“enlarged lymph nodes”).
- 纵隔、右肺门、双侧锁骨上区多发小淋巴结[there were multiple small lymph nodes in the mediastinum, right hilum, and bilateral supraclavicular area] = 小淋巴结(“small lymph nodes”).

Location

This type of entity refers to descriptions of location of suspicious masses and lymph nodes metastasis

Several typical examples are described in the following:

- 纵隔 2R、4、7 组多发肿大淋巴结[there were multiple enlarged lymph nodes in mediastinal 2R, 4, and 7 groups] = 纵隔 2R、4、7 组(“mediastinal 2R, 4, and 7 groups”).
- 右肺上叶前段可见一分叶状软组织密度结节[there were lobular soft tissue density nodules in the anterior segment of the right upper lobe] = 右肺上叶前段(“anterior segment of the right upper lobe”).

Shape

This type of entity refers to descriptions of shape and margin of suspicious masses

Several typical examples are described in the following:

- 右肺上叶前段可见一分叶状软组织密度结节[there were lobular soft tissue density nodule in the anterior segment of the right upper lobe] = 分叶状(“lobular”).
- 边缘可见毛刺[there were burrs on the edges] = 毛刺(“burrs”).

Size

This type of entity refers to descriptions of size of suspicious masses and lymph nodes masses

Several typical examples are described in the following:

- 肿物大小约为[the size of the tumor size was approximately 24×19mm] = 24×19mm(“24×19mm”).

- 淋巴结大小约为[the size of the lymph node was approximately 14×7mm] = 14×7mm(“14×7mm”).

Density

This type of entity refers to descriptions of density of suspicious masses

Several typical examples are described in the following:

- 左肺下叶基底段见磨玻璃密度结节[there were ground glass density nodules in the basal segment of the left lower lobe] = 磨玻璃密度(“ground glass density”).

- 右肺上叶前段可见一实性结节[there was a solid density nodule in the anterior segment of the right upper lobe] = 实性(“solid”).

Enhancement

This type of entity refers to descriptions of enhancement degree of suspicious masses

Several typical examples are described in the following:

- 增强扫描边缘可见轻度强化[slightly enhanced edges could be seen after enhanced scanning] = 轻度强化(“slightly enhanced”).

- 增强扫描边缘可见不均匀强化[heterogeneous enhanced edges could be seen after enhanced scanning] = 不均匀强化(“heterogeneous enhanced”)..

Effusion

This type of entity refers to descriptions of pleural effusion and pericardial effusion caused by the suspicious masses

Several typical examples are described in the following:

- 右侧胸腔见胸水征象[there was a sign of pleural effusion in the right chest cavity] = 胸胸水征象(“sign of pleural effusion”).

- 左侧胸腔见少量胸水 [there was a small amount of pleural effusion in the left chest cavity] = 少量胸水(“a small amount of pleural effusion”).

Negation

This type of entity refers to descriptions of negation

Several typical examples are described in the following:

- 纵隔 2R、4、7 组未见肿大淋巴结[there were no multiple enlarged lymph nodes in mediastinal 2R, 4, and 7 groups] = 未见(“no”).

- 两肺未见小结节[there were no nodules in both lungs] = 未见(“no”).

Bronchus

This type of entity refers to descriptions of bronchus invasion caused by the suspicious masses

Several typical examples are described in the following:

- 支气管旁见一肿物，管腔狭窄[a lump near the bronchi with a narrowed lumen] = 管腔狭窄(“narrowed lumen”).

- 支气管旁见一肿物，管腔闭塞[a lump near the bronchi with an occluded lumen] = 管腔闭塞(“occluded lumen”).

Pleura

This type of entity refers to descriptions of pleura invasion caused by the suspicious masses

Several typical examples are described in the following:

- 肿物与背侧胸膜相连[the mass was connected to the dorsal pleura] = 相连(“connected ”).

- 左侧胸腔胸膜粗糙[left side pleura was rough] = 粗糙(“rough”).

Vessel

This type of entity refers to descriptions of vessel invasion caused by the suspicious masses

Several typical examples are described in the following:

- 肿物包绕右下肺静脉[the mass surrounded the right lower pulmonary vein] = 包绕右下肺静脉(“surrounds the right lower pulmonary vein”).
- 结节包裹右肺静脉[the nodule wrapped right pulmonary vein] = 包裹右肺静脉(“wrapped right pulmonary vein”).

Vertebral Body

This type of entity refers to descriptions of vertebral body invasion caused by the suspicious masses

Several typical examples are described in the following:

- T11 椎体见混杂密度灶[see mixed-density lesion in T11 vertebral body] = T11 椎体见混杂密度灶(mixed-density lesion in T11 vertebral body).
- 胸 7，10 椎体见不规则低密度骨质破坏灶[see irregular low-density bone destruction in thoracic 7 and 10 vertebral body] = 胸 7，10 椎体见不规则低密度骨质破坏灶 (irregular low-density bone destruction in thoracic 7 and 10 vertebral body).

Pulmonary Atelectasis/Obstructive Pneumonitis Entity Annotation

This type of entity refers to descriptions of the pulmonary atelectasis or Obstructive Pneumonitis caused by the suspicious masses

Several typical examples are described in the following:

- 远端可见肺不张影[there were atelectasis shadows in the distal lung tissue] = 远端可见肺不张影(atelectasis shadows in the distal lung tissue).
- 远端肺组织内可见少许斑片影[there were a few patchy shadows in the distal lung tissue] = 远端肺组织内可见少许斑片影(a few patchy shadows in the distal lung tissue).
